# Evolution of the prevalence of obesity in the adult population in France, 2013–2016: the Constances study

**DOI:** 10.1038/s41598-021-93432-0

**Published:** 2021-07-08

**Authors:** Sébastien Czernichow, Adeline Renuy, Claire Rives-Lange, Claire Carette, Guillaume Airagnes, Emmanuel Wiernik, Anna Ozguler, Sofiane Kab, Marcel Goldberg, Marie Zins, Joane Matta

**Affiliations:** 1 Université de Paris, Paris, France; 2 Assistance Publique-Hôpitaux de Paris (AP-HP), Service de Nutrition, Centre Spécialisé Obésité, Hôpital Européen Georges Pompidou, Paris, 75015 France; 3 INSERM, UMR1153, Epidemiology and Biostatistics Sorbonne Paris Cité Center (CRESS), Paris, France; 4 INSERM, UMS011, Population-Based Epidemiologic Cohorts, 16 av. Paul Vaillant Couturier, 94800 Villejuif, France; 5 Assistance Publique-Hôpitaux de Paris (AP-HP), Centre d’Investigation Clinique INSERM 1418,, Hôpital Européen Georges Pompidou, Paris, France; 6 Department of Psychiatry and Addictology, AP-HP.Centre-Université de Paris, Paris, France

**Keywords:** Nutrition disorders, Obesity

## Abstract

This study provides trends in obesity prevalence in adults from 2013 to 2016 in France. 63,582 men and women from independent samples upon inclusion from the Constances cohort were included. Anthropometrics were measured at Health Screening Centers and obesity defined as a Body mass index (BMI) ≥ 30 kg/m^2^; obesity classes according to BMI are as follows: class 1 [30–34.9]; class 2 [35–39.9]; class 3 [≥ 40 kg/m^2^]. Linear trends across obesity classes by sex and age groups were examined in regression models and percentage point change from 2013 to 2016 for each age category calculated. All analyses accounted for sample weights for non-response, age and sex-calibrated to the French population. Prevalence of obesity ranged from 14.2 to 15.2% and from 14 to 15.3% in women and men respectively from 2013 to 2016. Class 1 obesity category prevalence was the only one to increase significantly across survey years in both men and women (*p* for linear trend = 0.04 and 0.01 in women and men respectively). The only significant increase for obesity was observed in the age group 18–29 y in both women and men (+ 2.71% and + 3.26% point increase respectively, equivalent to an approximate rise of 50% in women and 93% in men, *p* = 0.03 and 0.02 respectively). After adjustment for survey non-response and for age and sex distribution, the results show that class 1 obesity prevalence has significantly increased in both women and men from 2013 to 2016, and only in young adults in a representative sample of the French population aged 18–69 years old.

## Introduction

The prevalence of obesity has rapidly risen from the beginning of the 1980s, reaching the count of more than 2 billion people affected worldwide in 2015^1^. Obesity is an important risk factor for poor health such as cardiovascular diseases, type 2 diabetes, certain cancers, reduced life expectancy and mortality^2, 3^. Worldwide data based on 1698 studies has shown that the global prevalence of obesity has increased from 3.2 to 10.8% in men and from 6.4 to 14.9% in women between 1975 and 2014^1^.

The prevalence of severe obesity in the United States (US) was 9.2% among adults in 2017–2018, women having a higher prevalence of severe obesity (11.5%) compared to men (6.9%). Severe obesity prevalence was the highest among adults aged 40–59 (11.5%) compared to adults aged 60 years old and above (5.8%)^4^.

In France, few studies have documented the prevalence of obesity with estimates ranging from 15 to 17.2% among adults^5–7^. The Obepi study was conducted every three years from 1997 to 2012 and was based on self-reported weight and height. The estimated prevalence in 2012 was 15%, reflecting an increase of 76.4% from the period of 1997–2009, a figure that has tapered off thereafter.

The Esteban study conducted by Public Health France has estimated that the prevalence of obesity was 17.2% in adults aged 18–74 years of age (n = 3,000). This study has also highlighted that the prevalence of obesity increased with age in both sexes (21.5% and 20.6% in men and women aged 55–74 years of age versus 10.1% and 11.3% in men and women aged 18–39 years old, respectively)^6, 8^. Cross sectional data from the Constances cohort conducted in 2013 on participants aged 30–69 has found a prevalence of obesity of 15.8% in men and 15.6% in women. The cross-sectional estimates of obesity prevalence in the aforementioned studies are somehow coherent but less is known about trends in obesity and severe obesity (i.e., grades of obesity) in France while analyzing independent samples with the same methodology and selection criteria, as well as throughout different age groups and socio-economic backgrounds. Moreover, representative data adjusting for survey non-response and for age and sex distribution of the French population is lacking. The main objective of this paper is to present the most recent trends in obesity prevalence using standardised operational protocols for weight and height measurements for survey years 2013–2016 among adults in France from the Constances cohort^9^. The second objective is to study the prevalence of obesity by lifestyle factors such as smoking and physical activity as well as its association with income and education.

## Methods

### Population

Constances is a large, population-based, prospective cohort whose recruitment began in 2012 and ended in 2019; it involves a total of more than 200,000 subjects, including volunteers aged 18–69 years at baseline, and living in 21 selected departments from a total of 26 sub-centers, which were not randomly selected, throughout metropolitan France, in both rural and urban settings^10^.

Participants were selected among individuals covered by the general insurance scheme or partner health mutual societies (in all, 85% of the French population) using a random sampling scheme stratified on place of residence, age, gender, occupation and socioeconomic status. Eligible individuals were invited to participate in the study by mail. Volunteers completed a self-administered questionnaire on socio-professional status, and attended a Health Screening Center for a comprehensive evaluation including a physical examination and laboratory tests.

The Constances cohort study has received the authorization of the French Data Protection Authority (Commission Nationale de l’Informatique et des Libertés, CNIL) and the institutional review board of the National Institute for Medical Research (Inserm) (Authorization number 910486) and all methods were performed in accordance with the relevant guidelines and regulations. All subjects included in this study gave their informed consent.

**Figure 1:**
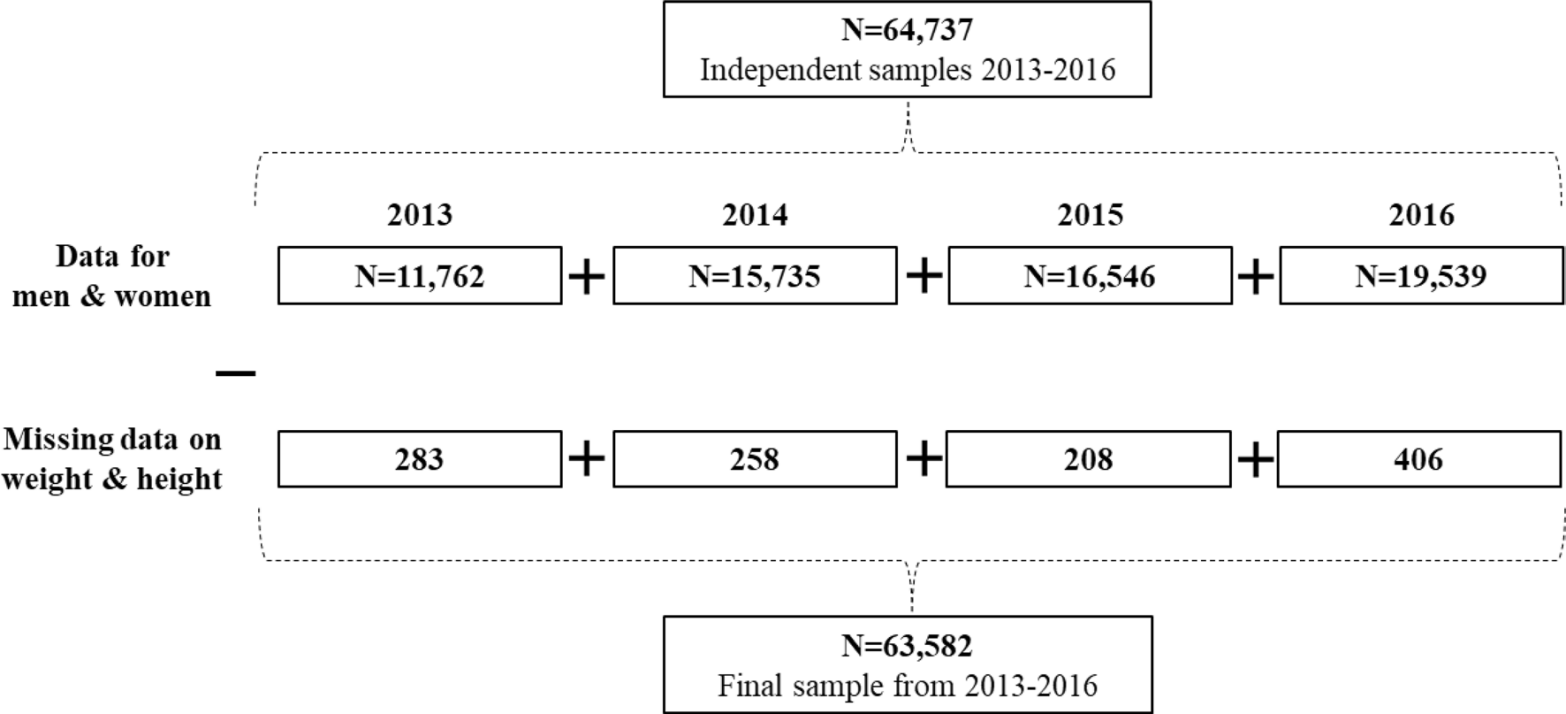
Flowchart for the selection of participants.

The present analyses were restricted to individuals with survey years 2013–2016, with complete baseline data on weight, height, age and sex, and were all affiliated to the General Social Security Regime. Pregnant women (N = 334) were excluded from the analyses. Details on participants’ selection by survey year (n = 63,582) are presented in Fig. 1.

### Variables

#### Anthropometrics

Weight and height were measured in Health Screening Centers. Weight was measured using a non-automatic weighing instrument based on the recommendation of the International Organisation of Legal Metrology (OIML R 76-1, Edition 2006). Standing height is measured with a fixed stadiometer with a vertical backboard and a moveable headboard to the nearest 0.1 cm.

To determine obesity status, BMI was calculated using the following formula: weight (kg)/height (m^2^) and four categories were determined according to the World Health Organization criteria: underweight [BMI < 18.5]; normal weight [18.5–24.9 kg/m^2^]; overweight BMI [25–29.9 kg/m^2^]; and individuals with obesity BMI ≥ 30 kg/m^2^. BMI was modeled as a categorical variable: underweight, normal weight, overweight and obese. Obesity classes were defined as follows: class 1 [30–34.9]; class 2 BMI [35–39.9]; class 3 BMI [≥ 40 kg/m^2^].

#### Waist circumference

Waist circumference was measured in cm with a tape measure and abdominal obesity was defined according to two definitions: High Authority of Health [≥ 94 cm in men and ≥ 80 cm in women]; and National Cholesterol Education Program (NCEP) [> 102 cm in men and > 88 cm in women]. All anthropometric measurements were obtained following standardized procedures^11, 12^.

### Socio demographic variables

The following variables were reported on a paper-based version baseline questionnaire.

#### Demographic

Age and sex were self-reported in the baseline questionnaire and age was further categorized into 5 groups: 18–29; 30–39; 40–49; 50–59; and 60–69 years.

#### Education

Education was measured using the following question: “What is the highest diploma that you have obtained?” Participants had to select among the following options: (1) no diploma; (2) general study certificate (equivalent to 12 years of school education); (3) certificate of professional aptitude; (4) high school diploma or equivalent; (5) undergraduate degree (2–3 years of study); (6) graduate degree (4 years of study); (7) graduate degree (≥ 5 years of study); or (8) other. Category 8 (other) has been combined with category 1 (no diploma) even though few individuals in category 8 had high diplomas but because of their small number they were not analyzed in a separate category.

#### Income

Income was measured by the following question: “What is the total amount of the net monthly income of your household? (i.e., the sum of all the incomes of people living in your household or your income if you live alone, regardless of the source of the income)?”. Participants had to select among the following options: (1) less than 450 euros; (2) 450 to less than 1000 euros; (3) 1000 to less than 1500 euros; (4) 1500 to less than 2100 euros; (5) 2100 to less than 2800 euros; (6) 2800–4200 euros; (7) more than or equal to 4200 euros; (8) don’t know how to answer; or (9) does not wish to answer. Categories 1 and 2 were further combined into one category (less than 1000 euros).

## Other variables

### Physical activity

Physical activity outside work was determined by a calculated score ranging from 0 (i.e., being not active at all) to 6 (being very active). Physical activity was further categorized into low (score of 0–2), medium (score of 3–4), and high (score of 5–6) level.

#### Smoking status

Smoking use (i.e., non-smoker, former smoker, or current smoker) was self-reported and smoking was modeled as a three category variable.

### Statistical analyses

Participants’ characteristics by survey year are presented as percentages and 95% CI. Prevalence and 95% CI of BMI categories and obesity were calculated by survey year and are presented by age group, sex, income, education and smoking status. Further stratification was made by obesity classes (classes 1, 2 and 3).

Linear trends across obesity classes by sex, age group, categories for education and income were examined in regression models with four time points (survey years 2013–2016) modelled as an orthogonal polynomial and *p* values were reported.

Logistic regression models, further adjusting for age, sex, physical activity and smoking status and analysed separately for education and income were conducted in survey year 2016 to determine if obesity prevalence could be explained by these factors. Since education and income are highly associated we have chosen to separate both outcomes to show their respective strength of their association with obesity.

The choice of survey year 2016 (the most recent available survey year) to conduct the logistic regressions was made because the prevalence of obesity by education and income status was consistent across survey years and trends of difference from 2013 to 2016 were non-significant (results not shown).

### Weighting and calibration

First, sample weights for non-response were performed using propensity scores in order to provide results representative of the French general population covered by the general health insurance scheme. A weighting coefficient has been computed for each subject by the CONSTANCES team. This coefficient took into account both the survey weight and the non-participation correction factor based on the follow-up of a control cohort of non-participants^10, 13^. The weights consider the variables that are associated to the probability of participation to the Constances cohort such as (but not limited to) sociodemographics, socio-professional categories, healthcare use and hospitalisations.

Second, we further calibrate for age and sex using the National Institute of Statistics and Economic Studies (Institut national de la statistique et des études économiques, INSEE) 2016 data which was performed by the ‘raking ratio’ method executed by proc Calmar in SAS. Independent samples upon inclusion to the Constances cohort from 2013 to 2016 were selected.

Age and sex distributions in the Constances study versus those of the French population are presented.

Tabular estimates for the prevalence of BMI categories (i.e., underweight, normal weight, overweight and obese) and obesity classes 1, 2 and 3 are presented by survey years, sex and age groups.

Analyses were conducted using SAS (SAS Institute) version 9.4. A 2-sided *p* value of 0.05 was used to assess statistical significance.

## Results

### Baseline characteristics

A total of 65,071 subjects affiliated to the General Social Security Regime from the Constances cohort were selected from survey years 2013–2016 (Fig. 1) and pregnant women (N = 334) were excluded. Among the selected sample, 1155 (1.8%) subjects had missing data on height and weight. Comparison of survey sample in the Constances study with the general French population is presented in Supplementary Table 1. The final sample is based on 63,582 subjects (Table 1) with half of the population being female, mean (SD) age was 44.4 (0.1) and 46.2 (0.1) years in women and men, respectively. About 65% of the population had baccalaureate degree or higher, a quarter was active smokers and a third had a low physical activity level. Abdominal obesity (≥ 88/102 cm, in women and men) ranged from 24 to 26% in women and from 17.2 to 20% of men.

**Table 1:** Participants’ characteristics by survey year (N = 63,582).

	2013	2014	2015	2016
	% (95 CI)			
**Sex**				
Women	50.12 (48.7–51.4)	50.9 (49.8–52.0)	50.7 (49.6–51.9)	51.2 (50.2–52.2)
**Age group (years)**				
18–29	17.0 (15.9–18.2)	17.9 (17.0–18.8)	15.0 (14.2–15.8)	15.7 (14.9–16.4)
30–39	22.6 (21.4–23.8)	23.0 (22.1–24.0)	23.0 (22.1–24.0)	21.5 (20.7–22.3)
40–49	22.0 (20.9–23.0)	22.8 (21.9–23.7)	22.1 (21.2–23.0)	20.6 (19.8–21.4)
50–59	19.8 (18.8–20.9)	19.1 (18.3–19.9)	19.8 (18.9–20.7)	18.5 (17.7–19.3)
60–69	18.3 (17.4–19.3)	17.0 (16.2–17.8)	19.8 (18.9–20.8)	23.5 (22.5–24.4)
**Waist circumference ≥ 94 cm for men and ≥ 80 cm for women (missing = 142)**				
Women	43.8 (41.9–45.7)	44.6 (43.1–46.1)	45.4 (43.9–46.9)	45.6 (44.3–47.0)
Men	36.1 (34.3–38.0)	34.9 (33.5–36.4)	39.0 (37.4–40.6)	39.6 (38.1–41.1)
**Waist circumference ≥ 102 cm for men and ≥ 88 cm for women (missing = 142)**				
Women	24.1 (22.4–25.8)	26.0 (24.6–27.4)	24.5 (23.2–25.9)	25.9 (24.6–27.1)
Men	17.2 (15.7–18.6)	16.5 (15.4–17.7)	19.0 (17.6–20.3)	19.9 (18.6–21.2)
**Education (missing = 1445)**				
No diploma or other	4.9 (4.2–5.6)	5.3 (4.7–5.8)	5.4 (4.8–5.9)	4.3 (3.8–4.8)
General education certificate, Primary education certificate, School-leaving certificate	7.6 (6.8–8.3)	6.7 (6.2–7.3)	6.9 (6.3–7.5)	7.2 (6.6–7.7)
Certificate of professional competence, vocational training certificate	21.3 (20.1–22.5)	21.2 (20.3–22.2)	21.0 (19.9–21.8)	19.8 (18.9–20.7)
Baccalaureate or equivalent diploma	17.5 (16.4–18.5)	18.4 (17.5–19.2)	17.5 (16.7–18.4)	17.2 (16.4–18.0)
Baccalaureate + 2 or 3 years	23.9 (22.8–25.1)	23.8 (22.9–24.7)	23.1 (22.1–24.0)	24.5 (23.7–25.4)
Baccalaureate + 4 years	5.9 (5.3–6.5)	5.5 (5.1–6.0)	5.9 (5.4–6.4)	5.5 (5.1–5.9)
Baccalaureate + 5 years and more	18.5 (17.6–19.5)	18.7 (17.9–19.5)	19.9 (19.0–20.7)	21.2 (20.4–22.0)
**Income**				
Less than 1000 €	11.9 (10.9–13.0)	12.1 (11.3–12.9)	11.2 (10.4–12.0)	10.1 (9.4–10.8)
From 1000 € to less than 1500 €	10.0 (9.1–10.9)	10.8 (10.1–11.6)	10.2 (9.4–10.9)	9.4 (8.7–10.0)
From 1500 € to less than 2100 €	13.8 (12.8–14.8)	13.1 (12.4–13.9)	12.5 (11.7–13.2)	12.7 (12.0–13.4)
From 2100 € to less than 2800 €	15.5 (14.6–16.5)	16.0 (15.2–16.8)	15.7 (14.9–16.6)	15.2 (14.4–15.9)
From 2800€ to 4200€	25.0 (23.9–26.1)	25.1 (24.2–26.0)	25.8 (24.9–26.8)	27.2 (26.4–28.1)
4200€ and over	17.7 (16.8–18.6)	17.3 (16.6–18.0)	18.7 (17.9–19.5)	19.8 (19.0–20.5)
Don't know the answer	1.3 (0.9–1.7)	1.4 (1.1–1.7)	1.1 (0.9–1.4)	1.3 (1.0–1.5)
Don't want to answer	4.3 (3.8–4.8)	3.7 (3.4–4.1)	4.3 (3.9–4.8)	4.0 (3.6–4.5)
**Smoking status (missing = 3207)**				
Non-smokers	43.9 (42.5–45.2)	44.3 (43.2–45.4)	44.4 (43.3–45.5)	45.5 (44.4–46.5)
Smokers	24.9 (23.6–26.2)	25.1 (24.1–26.1)	23.6 (22.6–24.6)	21.7 (20.8–22.6)
Ex-smokers	31.0 (29.8–32.3)	30.5 (29.5–31.5)	31.8 (30.8–32.9)	32.7 (31.7–33.7)
**Physical activity level (missing = 2617)**				
Low	32.4 (31.0–33.7)	32.8 (31.8–33.9)	31.4 (30.3–32.4)	30.2 (29.2–31.2)
Moderate	45.4 (44.0–46.7)	45.2 (44.2–46.3)	45.1 (43.9–46.2)	45.1 (44.1–46.1)
High	22.1 (21.0–23.2)	21.8 (20.9–22.7)	23.4 (22.5–24.4)	24.6 (23.7–25.4)

Estimates of prevalence are weighted at year of invitation to survey participation.

Age and sex-adjusted by the raking ratio method using the Insee data and age groups 18–29, 30–39, 40–49, 50–59, and 60–69 years.

### Main findings

**Figure 2:**
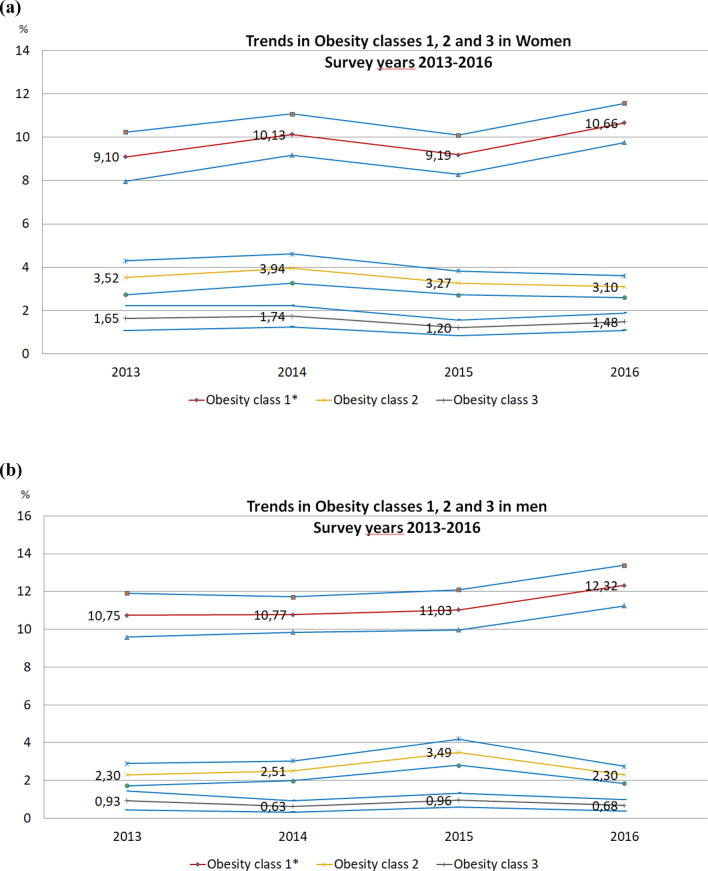
(**a**) Trends in obesity classes 1, 2 and 3 prevalence in women. Survey years 2013–2016. *Significant linear trend. P for trend estimated using the coefficients of orthogonal polynomials corresponding to linear contrast. P for linear trend for obesity grade 1 = 0.04 (F = 3.96), for obesity grade 2 = 0.27 (F = 1.20) and obesity grade 3 = 0.41 (F = 0.66). Age and sex-adjusted by the raking ratio method using the Insee data and age groups 18–29, 30–39, 40–49, 50–59, and 60–69 years. Dashed lines are confidence intervals. (**b**) Trends in obesity classes 1, 2 and 3 prevalence in men. Survey years 2013–2016. *Significant linear trend. P for trend estimated using the coefficients of orthogonal polynomials corresponding to linear contrast. P for linear trend for obesity grade 1 = 0.01 (F = 5.59), for obesity grade 2 = 0.32 (F = 0.97) and obesity grade 3 = 0.15 (F = 0.69). Age and sex-adjusted by the raking ratio method using the Insee data and age groups 18–29, 30–39, 40–49, 50–59, and 60–69 years. Dashed lines are confidence intervals.

**Figure 3:**
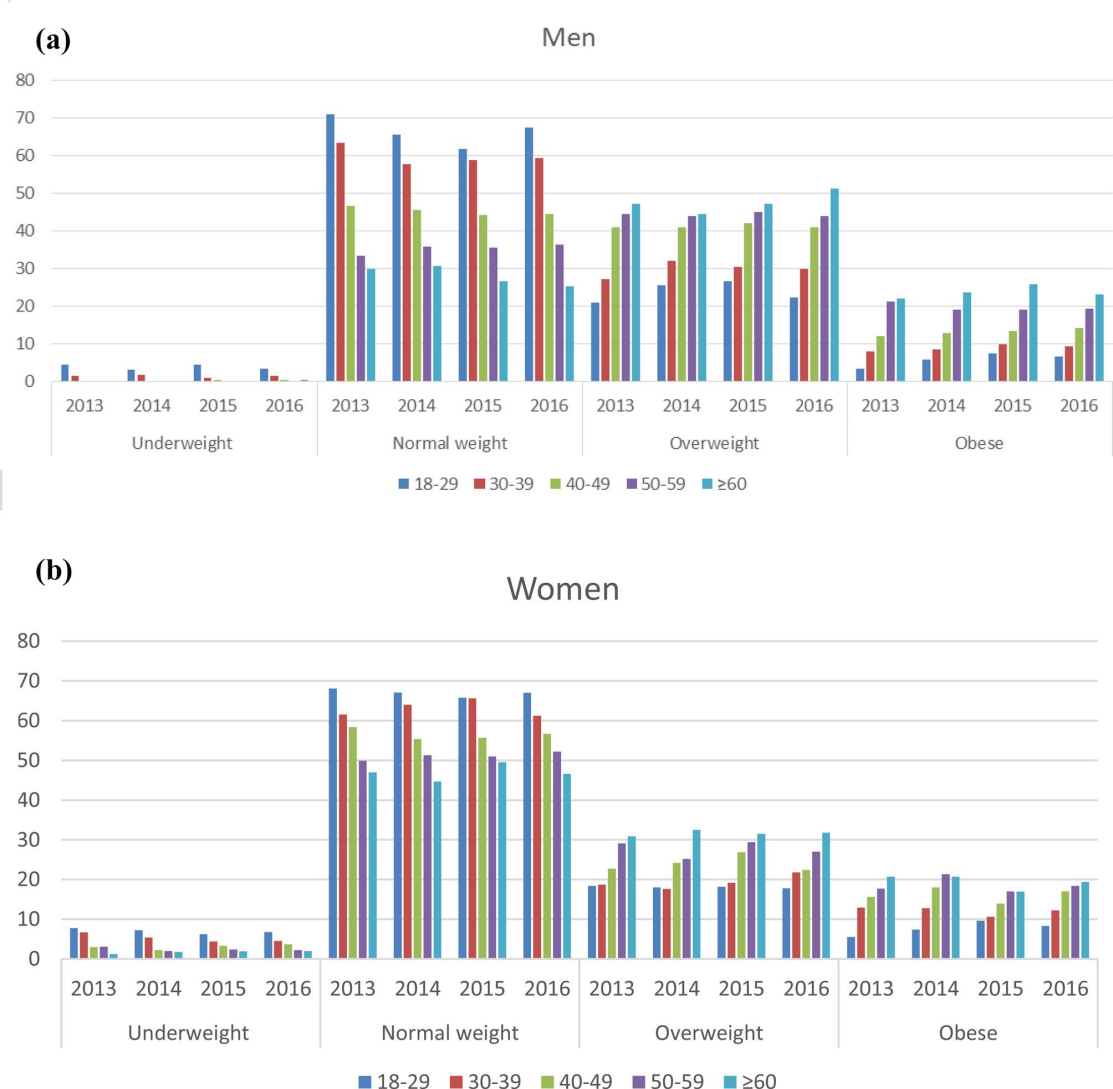
(**a**) BMI categories by age and survey year in men (N = 30,783). Empty bars indicate a prevalence of less than 10 cases and are not represented. Age and sex-adjusted by the raking ratio method using the Insee data and age groups 18–29, 30–39, 40–49, 50–59, and 60–69 years. (**b**) BMI categories by age and survey year in women (N = 32,799). Age and sex-adjusted by the raking ratio method using the Insee data and age groups 18–29, 30–39, 40–49, 50–59, and 60–69 years.

Prevalence of obesity ranged from 14.2% and 14% in women and men respectively in 2013 to 15.2% and 15.3% in 2016 (Fig. 2a,b). The trend for class 1 obesity category prevalence across survey years in both women and men was significant (*p* value for trend 0.04 and 0.01 respectively. Figure 3a,b show data of BMI categories by age groups and survey years, separately in men and women. In men, age and sex-adjusted estimates indicate that the prevalence of overweight and obesity prevalence in the 18–29 year-old category ranged from 24.4% in 2013 to 28.9% in 2016 and from 51.6 to 74.1% from 2013 to 2016 in participants ≥ 60 years old. In women, overweight and obesity prevalence from 2013 to 2016 ranged from 24.5 to 26.3% in the 18–29 year-olds and from 51.6 to 51.1% in individuals of 60–69 years old (all estimates are presented in Supplementary Table 2).

**Figure 4:**
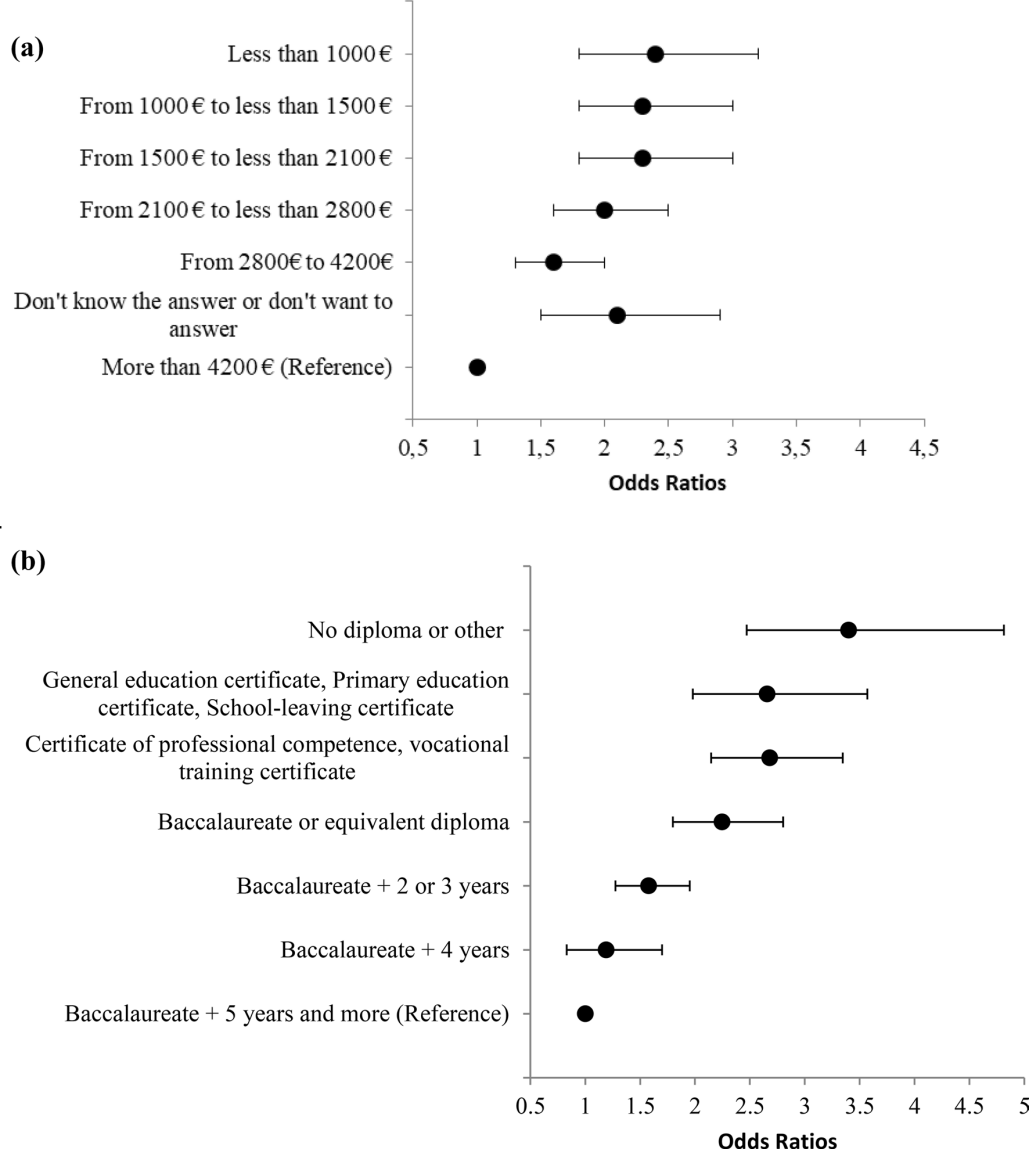
(**a**) ORs and 95% of obesity by income categories using multivariate logistic regression with weighted and age and sex-adjusted estimates in survey year 2016. Model adjusted for age, sex, smoking and physical activity level. Interaction of survey year was tested with each characteristic in the model and was not significant. Linear trend across income characteristic is significant (p < 0.0001). Age and sex-adjusted by the raking ratio method using the Insee data and age groups 18–29, 30–39, 40–49, 50–59, and 60–69 years. (**b**) ORs and 95% of obesity by education categories using multivariate logistic regression with weighted and age and sex-adjusted estimates in survey year 2016. Model adjusted for age, sex, smoking and physical activity level. Interaction of survey year was tested with each characteristic in the model and was not significant. Linear trend across education characteristic is significant (p < 0.0001). Age and sex-adjusted by the raking ratio method using the Insee data and age groups 18–29, 30–39, 40–49, 50–59, and 60–69 years.

Tables 2 and 3 indicate the prevalence of obesity by sex by and age groups according to survey year. An increasing gradient across age groups was observed at every survey year. From 2013 to 2016, the only significant linear trends was observed in the age group 18–29 in both women and men (*p* value for trend = 0.01), as well as a significant increase of + 2.71% and 3.26% point, equivalent to a 50% and 93% rise in obesity prevalence in women and men respectively, *p* for change from 2013 to 2016 = 0.03 and 0.02). The other age categories did not reach statistical significance neither for linear trends across survey years or point change between 2013 and 2016 in both sexes. Prevalence of obesity according to baseline characteristics and survey year is described in Table 4. Smokers had lower obesity prevalence compared to ex-smokers and physically active individuals had the lowest prevalence of obesity. Irrespective of the survey year, a strong decreasing gradient was observed with both education and income levels. As an example, for survey year 2016, the prevalence of obesity for those with no diploma was 29.5%, whilst those with a baccalaureate plus 5 years of education and more exhibited 7.3% prevalence. ORs and 95% CI of obesity in individuals with a monthly income lower than 1000 euros were 2.4 (1.8–3.2) compared to those with an income above 4200 euros and 3.4 (2.4–4.8) in individuals without a diploma compared to those having a graduate degree of more than 5 years of study, these results are presented in Fig. 4a,b.

**Table 2:** Linear trend of obesity by age, weighted and age and sex-adjusted prevalence, 2013–2016 in women (N = 32,799).

	Adult obesity^a^ % (95 CI)						
	2013 N = 5966	2014 N = 8223	2015 N = 8468	2016 N = 10,142	Points of change from 2013 to 2016^b^	*p* value of change from 2013 to 2016^c^	*p* value of linear trend test^d^
18–29 (N = 4690)	5.59 (3.70–7.48)	7.46 (5.48–9.43)	9.65 (7.32–11.97)	8.30 (6.44–10.16)	2.71 (0.06–5.36)	0.037	0.017
30–39 (N = 7235)	12.95 (9.92–15.99)	12.89 (10.50–15.28)	10.64 (8.46–12.82)	12.27 (10.35–14.19)	− 0.68 (–4.27–2.91)	0.70	0.45
40–49 (N = 7815)	15.69 (12.55–18.82)	18.07 (15.62–20.52)	13.96 (11.68–16.24)	17.00 (14.68–19.31)	1.31 (− 2.59–5.20)	0.51	0.97
50–59 (N = 7067)	17.75 (14.34–21.16)	21.34 (18.30–24.38)	17.04 (14.57–19.50)	18.44 (15.80–21.08)	0.69 (− 3.63–5.01)	0.75	0.74
60–69 (N = 5992)	20.77 (16.90–24.65)	20.76 (17.49–24.02)	16.99 (14.28–19.69)	19.46 (16.75–22.17)	− 1.31 (− 6.04–3.42)	0.58	0.30

^a^Adult obesity defined as BMI ≥ 30 kg/m^2^.

^b^Percentage point.

^c^From the t-test of the change from 2013 to 2016.

^d^From the coefficients of orthogonal polynomials corresponding to linear contrast.

Age and sex-adjusted by the raking ratio method using the Insee data and age groups 18–29, 30–39, 40–49, 50–59, and 60–69 years.

**Table 3:** Linear trend of obesity by age, weighted and age and sex-adjusted prevalence, 2013–2016 in men (N = 30,783).

	Adult obesity^a^ % (95 CI)						
	2013 N = 5796	2014 N = 7512	2015 N = 8078	2016 N = 9397	Points of change from 2013 to 2016^b^	*p* value of change from 2013 to 2016^c^	*p* value of linear trend test^d^
18–29 (N = 3775)	3.49 (1.25–5.73)	5.72 (3.75–7.69)	7.30 (5.05–9.56)	6.75 (4.99–8.51)	3.26 (0.41–6.12)	0.024	0.013
30–39 (N = 6504)	8.04 (5.58–10.50)	8.62 (6.81–10.43)	9.75 (7.62–11.88)	9.21 (7.17–11.25)	1.17 (− 2.03–4.37)	0.47	0.36
40–49 (N = 7360)	12.11 (9.53–14.68)	12.84 (10.72–14.95)	13.37 (11.01–15.73)	14.13 (12.00–16.27)	2.03 (− 1.32–5.37)	0.23	0.21
50–59 (N = 6608)	21.31 (17.91–24.70)	19.08 (16.25–21.87)	19.17 (16.18–22.16)	19.30 (16.50–22.09)	–2.01 (− 6.42–2.39)	0.36	0.39
60–69 (N = 6536)	22.09 (18.74–25.44)	23.77 (20.71–26.83)	25.94 (22.40–29.48)	23.03 (20.02–26.04)	0.94 (− 3.57–5.44)	0.68	0.49

^a^Adult obesity defined as BMI ≥ 30 kg/m^2^.

^b^Percentage point.

^c^From the t-test of the change from 2013 to 2016.

^d^From the coefficients of orthogonal polynomials corresponding to linear contrast.

Age and sex-adjusted by the raking ratio method using the Insee data and age groups 18–29, 30–39, 40–49, 50–59, and 60–69 years.

**Table 4:** Prevalence of obesity by income, education, smoking and physical activity level (N = 63,582).

Characteristic	Survey year			
	2013	2014	2015	2016
	% (95 CI)			
**Education (missing = 1445)**				
No diploma or other	26.7 (20.6–32.9)	26.8 (21.8–31.8)	20.1 (15.8–24.5)	29.6 (24.5–34.8)
General education certificate, Primary education certificate, School–leaving certificate	24.2 (19.7–28.7)	26.3 (22.3–30.2)	22.1 (18.4–25.8)	23.5 (19.8–27.3)
Certificate of professional competence, vocational training certificate	20.9 (18.3–23.5)	20.0 (18.0–22.0)	20.6 (18.4–22.8)	22.0 (19.8–24.2)
Baccalaureate or equivalent diploma	14.3 (11.9–16.8)	14.2 (12.4–16.0)	13.8 (11.8–15.7)	16.4 (14.4–18.3)
Baccalaureate + 2 or 3 years	10.1 (8.4–11.8)	10.8 (9.4–12.2)	12.0 (10.5–13.5)	11.8 (10.5–13.1)
Baccalaureate + 4 years	7.7 (5.1–10.4)	10.8 (8.3–13.9)	13.1 (9.7–16.6)	9.6 (7.1–12.2)
Baccalaureate + 5 years and more	5.4 (4.3–6.6)	7.5 (6.2–8.8)	7.5 (6.1–8.8)	7.4 (6.3–8.5)
**Income**				
Less than 1000 €	19.7 (15.9–23.6)	19.7 (16.7–22.8)	16.8 (14.0–19.6)	19.4 (16.4–22.4)
From 1000 € to less than 1500 €	18.5 (14.8–22.2)	18.2 (15.4–21.0)	18.9 (15.8–21.9)	19.3 (16.4–22.2)
From 1500 € to less than 2100 €	14.5 (11.8–17.2)	16.9 (14.6–19.3)	14.4 (12.1–16.8)	19.1 (16.7–21.6)
From 2100 € to less than 2800 €	14.6 (12.3–16.8)	16.1 (14.1–18.1)	17.6 (15.2–20.0)	16.7 (14.7–18.7)
From 2800€ to 4200€	13.4 (11.6–15.2)	11.8 (10.5–13.1)	12.9 (11.5–14.3)	13.6 (12.2–15.0)
Don't know the answer	Less than 10 cases	22.6 (13.4–31.8)	14.2 (6.4–22.0)	21.5 (13.4–29.6)
Don't want to answer	12.2 (8.2–16.1)	16.5 (12.1–20.8)	20.8 (16.5–25.1)	16.8 (12.8–20.9)
4200€ and over	9.0 (7.3–10.6)	10.0 (8.5–11.4)	9.0 (7.6–10.4)	9.1 (7.8–10.3)
**Smoking status (missing = 3207)**				
Non-smokers	13.9 (12.4–15.4)	14.5 (13.3–15.8)	13.6 (12.4–14.9)	13.7 (12.6–14.8)
Smokers	10.5 (8.6–12.4)	11.3 (9.7–12.9)	10.5 (9.0–11.9)	11.2 (9.7–12.7)
Ex-smokers	16.1 (14.4–17.9)	17.7 (16.1–19.2)	18.3 (16.6–20.0)	19.5 (17.9–21.1)
**Physical activity level (missing = 2617)**				
Low	17.9 (15.8–19.9)	20.0 (18.4–21.6)	18.7 (17.0–20.4)	19.6 (18.0–21.2)
Moderate	13.2 (11.8–14.6)	12.6 (11.5–13.7)	13.3 (12.1–14.5)	14.2 (13.0–15.4)
High	9.0 (7.4–10.6)	9.9 (8.5–11.4)	11.1 (9.6–12.7)	10.6 (9.2–12.0)

Age and sex-adjusted by the raking ratio method using the Insee data and age groups 18–29, 30–39, 40–49, 50–59, and 60–69 years.

Linear trends in abdominal obesity prevalence by age groups and sex are presented in Supplementary Tables 3a and 3b.

## Discussion

We took advantage of the Constances cohort to surface linear trends of obesity prevalence in independent samples through survey years 2013–2016.

In the present analyses, class 1 obesity prevalence increased in both men and women (9.1–10.6% in women and 10.7–12.3% in men) from 2013 to 2016, whereas the other obesity classes (2 and 3) remain relatively stable across this time span in both sexes.

In addition, we have determined linear trends of obesity by sex across age groups; in these subgroup analyses, linear trends have only significantly increased in the youngest adults (i.e., 18–29 years of age) in both women and men. We have not observed any significant changes across the other age groups. The rising trends in obesity found in Constances from 2013 to 2016, even though only significant for obesity class 1 and for young adults; do not show a massive rise in global obesity similar to that seen from 1997 to 2009 in Obepi (which had shown an increase of 76.4%)^5, 14^. It should be noted however that weight and height data have been declared in the French Obepi study compared to Constances, this may have led to underestimation in obesity prevalence in Obepi which varies with age, sex and period. Our results are more comparable to the Esteban study in France, which is based on measured anthropometrics and shows that from 2006 to 2015, obesity prevalence has been relatively stable at around 17% in adults’ men and women. In fact, data from several countries show a decline or stabilization of obesity levels, albeit with a mixed evidence, data from 2015 to 2016 prevalence indicating no evidence of a decline in obesity at any age^15^.

Recent data from the United States has shown that although from 1999–2000 through 2017–2018, the prevalence of global obesity and also severe obesity have increased, the most recent period of interests between 2015–2016 and 2017–2018 did not show a significant increase (5). It remains that we have observed a significant increase in individuals with obesity class 1, but not in individuals with obesity classes 2 and 3.

We have also found a 50% increase in women and 93% increase in men young adults. This finding is not consistent with data from the United States that shows no evidence for an increase in obesity in 2015–2016 versus 2017–2018 among individuals aged 20–39 years old, the prevalence being much higher than that in France (40%) which might suggest a slight tapering off at increased levels of obesity in this age group in the United States^4^. In the Constances cohort, even if the latest prevalence in this subgroup is not very high (7.7%), such an increase over a four year period is worrying. Our findings indicate that young adults should not be overlooked in health-policy making with regard to obesity. Since obesity is higher in low income statuses and the increase in poverty across years in the young groups may explain our finding, we have looked at the prevalence of different income categories across survey years, but have not found an increase in the prevalence of low income groups (results not shown).

We have further analyzed linear trends of obesity in young individuals among employees and students, and found out that obesity increased significantly in young employees (*p* for trend < 0.0001) but not students. This additionally highlights the social inequalities with regard to obesity as students who have access to education are less likely to be affected by this increase. This is also in line with our finding on the higher prevalence of obesity among individuals with lower education and income. Although the reason behind such an increase among young individuals and no other remains to be determined, the result of such an increase in Constances could be attributed to peer social transmission and non-social mechanisms. Researchers studying dynamic simulation models to understand how underlying population age structure and different mechanisms influence trends of obesity, have shown that obesity prevalence was most sensitive to adult parameters (such as social interactions, interplay of individual behaviors and norms)^15^. Our results show a somehow lower prevalence of obesity in the four study points compared to the Esteban study which was also based on measured anthropometrics and has found a prevalence of obesity of 17% in adults aged 18–74 years. Our study is consistent with Esteban in that it shows a higher prevalence of obesity in the 40–69 age groups compared to younger adults (18–39).In addition, we have observed a significant higher prevalence of obesity among individuals with lower education or income, even after adjustment for age, sex, physical activity and smoking status in multivariate models. It should be noted however that income was not measured per unit of consumption but rather as net monthly revenue for the household. Income in our analyses was measured through a range of pre-specified categories and we were able to find a linear trend of increase in the prevalence of obesity going from high to lower income categories. This is consistent with our previous finding in the Constances cohort among 30–69 year-old participants (15). To further test whether obesity prevalence has increased among different income or education categories, we have tested linear trends from 2013 to 2016 and have observed that results are not significant (results not shown herein); in other words obesity prevalence has not changed significantly in the same category of income or education across survey years; thus obesity remains high in the most financially deprived individuals and lower in the more privileged participants.

Our finding of higher obesity prevalence among lower education and income groups is consistent with other studies. Results from the French Abena study have shown that adult women relying on food aid programs had two times more obesity prevalence compared to the general population (35% vs. 15%)^16^. In the United States during 2011–2014, the age-adjusted prevalence of obesity among adults was lower in the highest income group (31.2%) than the other groups and the age-adjusted prevalence of obesity among college graduates was lower (27.9%) than among those with some college (40.6%)^17^.

One reason for the association between low education and income and obesity may be psychosocial stress and this exposure may mediate the relationship between poverty and increased obesity risk^18^. Additionally, low income may be associated with an increased risk of obesity by the limited access to healthy food and the toxic environment leading to low physical activity levels^18^. This highlights the relevance of public health programs to focus on healthier environments for the reduction of obesity prevalence among the most deprived individuals.

Interestingly, we have found a significant linear trend in older adults men (60–69 years) for abdominal obesity in men (waist circumference ≥ 94 cm) but not in women. Although, obesity rates have not increased significantly in this subgroup, adults aged 60–69 years old are at risk of developing abdominal obesity.

## Strengths and limitations

The strengths of our study include the large sample and the design of the Constances cohort that have allowed us to study trends in obesity prevalence by selecting independent samples of participants upon inclusion. All anthropometric data have been collected at Health Screening Centers (HSC) in France and routine protocols and checkups have been conducted by specialized teams^12^. Moreover, the calculated weightings for non-response that are taken into consideration with such complex survey design, have allowed us to render the analyzed sample representative of the Constances’ population and all estimates were age and sex-adjusted according to the INSEE data. Moreover, to our knowledge, this study provides recent trends with several time points of obesity prevalence in France.

Limitations include the low number of survey years included in our analyses (4 years). These four time points were able to provide a statistical estimation of a linear trend, however we are cognizant that more time points are needed to provide a better estimation of this linear variation. Additionally, the results are representative of the population affiliated to the General Social Security Regime and aged between 18–69 years old and cannot be representative of the French population. We have used the INSEE data to adjust for the age and sex distribution of the French population but are cognizant that this may differ from the Constances’ cohort population”, particularly because certain socio professional categories (with a low prevalence in our sample) are not represented in Constances, such as independent workers, farmers and craftsmen.

Our findings of an increase in the obesity class 1 category, and among the 18–29 age group, need to be interpreted with caution, as it is important to note that analysis of time trends depends on the initial point of examination (16). We have selected the first year of inclusion of participants in Constances as a starting point (2013); however a comparison between different starting time points may lead to different interpretations.

## Conclusion

Overall, there have been significant changes in obesity prevalence in the obesity class 1 category in both men and women. Obesity classes 2 and 3 remained stable across survey years. An increase of 50% and 93% (women and men) in obesity prevalence was found in young adults aged 18–29 years between 2013–2016, whereas it did not reach significance in the other age subgroups. Obesity prevalence remained high in the most socially deprived or less educated individuals. More survey points are needed to draw conclusions about the rising trends of obesity in France, whilst it remains important to monitor its prevalence.

## Supplementary Information

Supplementary Information.

## Data Availability

The data that support the findings of this study are available from the corresponding author upon reasonable request.
