# Supplementary material for: Evolution of the prevalence of obesity in the adult population in France, 2013–2016: the Constances study
Source: Sci Rep. 2021 Jul 8;11:14152. doi: 10.1038/s41598-021-93432-0 (PMC8266816; doi:10.1038/s41598-021-93432-0)
Supplement: Supplementary file 1 — Supplementary Information. [file 41598_2021_93432_MOESM1_ESM.docx]

**Supplementary table 1. Age and sex distribution in Constances and the French population (18-75 years old)**

|  | **Constances*** | **Insee (French population)** |
| --- | --- | --- |
| **Sex** |  |  |
| Men | 30783 (48.1) | 22499400 (48.8) |
| Women | 33129 (51.8) | 23576237 (51.1) |
| **Age groups** |  |  |
| 18-29 | 8565 (13.4) | 9276956 (20.1) |
| 30-39 | 13944 (21.8) | 8191538 (17.7) |
| 40-49 | 15192 (23.7) | 8895252 (19.3) |
| 50-59 | 13680 (21.4) | 8716807 (18.9) |
| 60-69 | 12531 (19.6) | 10995084 (23.8) |

**Constances estimates are weighted for non-response and are those calculated before standardization*

*Estimates are N(%)*

**Supplementary table 2**. **Prevalence of underweight, normal weight, overweight and obesity in adults by sex, age and survey year (N=63,912)**

|  | **Men** | | | | | **Women** | | | | |
| --- | --- | --- | --- | --- | --- | --- | --- | --- | --- | --- |
|  | 18-29 | 30-39 | 40-49 | 50-59 | 60-69 | 18-29 | 30-39 | 40-49 | 50-59 | 60-69 |
|  | % (95 CI) | | | | | | | | | |
| **Underweight** |  |  |  |  |  |  |  |  |  |  |
| 2013 | 4.4 (1.8-7) | 1.4 (0.2-2.6) | Less than 10 cases | Less than 10 cases | Less than 10 cases | 7.8 (5.0-10.6) | 6.7 (4.7-8.7) | 3.0 (1.6-4.4) | 3.1 (1.4-4.8) | 1.2 (0.5-1.8) |
| 2014 | 3.1 (1.7-4.6) | 1.7 (0.9-2.4) | Less than 10 cases | Less than 10 cases | Less than 10 cases | 7.2 (5.4-9.1) | 5.3 (4.0-6.9) | 2.2 (1.3-3.1) | 2.0 (1.2-2.8) | 1.8 (1-2.7) |
| 2015 | 4.3 (2.4-6) | 1.0 (0.4-1.5) | 0.4 (0.08-0.8) | Less than 10 cases | Less than 10 cases | 6.2 (4.5-7.8) | 4.4 (3.2-5.6) | 3.2 (2.1-4.4) | 2.4 (1.5-3.4) | 1.9 (0.9-2.8) |
| 2016 | 3.4 (2.2-4.6) | 1.4 (0.7-1.9) | 0.5 (0.1-0.9) | Less than 10 cases | 0.5 (0.1-1) | 6.8 (5.1-8.6) | 4.5 (3.5-5.4) | 3.7 (2.6-4.8) | 2.2 (1.3-3.1) | 2.0 (1.2-2.8) |
| **Normal weight** |  |  |  |  |  |  |  |  |  |  |
| 2013 | 71.0 (65.5-76.4) | 63.2 (58.6-67.3) | 46.5 (42.5-50.2) | 33.4 (29.5-36.8) | 29.8 (26.4-33) | 68.1 (63.5-72.8) | 61.5 (57.4-65.5) | 58.4 (54.7-62.2) | 49.9 (46-54) | 47.0 (42.9-51.1) |
| 2014 | 65.6 (61.4-69.5) | 57.6 (54.2-60.7) | 45.6 (42.2-48.6) | 35.8 (32.5-39) | 30.6 (27.4-33.9) | 67.1 (63.6-70.7) | 64.0 (60.8-67.1) | 55.4 (52.4-58.3) | 51.3 (48-54.5) | 44.7 (41.2-48.3) |
| 2015 | 61.7 (57.4-66.1) | 58.7 (55.6-62) | 44.1 (40.8-47.4) | 35.4 (32-38.8) | 26.6 (23.2-29.9) | 65.8 (62.1-69.5) | 65.6 (62.1-68.7) | 55.7 (52.6-58.8) | 51.0 (48-54.3) | 49.5 (45.9-53.1) |
| 2016 | 67.5 (63.3-70.8) | 59.4 (56.5-62.4) | 44.4 (41.3-47.2) | 36.5 (33.1-39.7) | 25.2 (22.3-27.9) | 67.0 (63.6-70.3) | 61.2 (58.6-63.9) | 56.7 (53.9-59.5) | 52.2 (49.1-55.3) | 46.6 (43.3-49.9) |
| **Overweight** |  |  |  |  |  |  |  |  |  |  |
| 2013 | 21.0 (16.1-25.9) | 27.2 (23.1-31) | 41.1 (37.3-44.9) | 44.4 (40.1-48) | 47.1 (43.2-50.9) | 18.4 (14.4-22.3) | 18.7 (15.5-21.9) | 22.7 (19.6-25.9) | 29.1 (25.3-32.8) | 30.9 (27-34.8) |
| 2014 | 25.4 (21.6-29.1) | 32.0 (28.8-34.9) | 41.0 (37.7-44.1) | 44.0 (40.6-47.3) | 44.5 (41-48.1) | 18.0 (15.1-20.9) | 17.6 (15.1-20.0) | 24.2 (21.6-26.8) | 25.2 (22.4-28) | 32.5 (29.1-35.9) |
| 2015 | 26.6 (22.5-30.7) | 30.4 (27.3-33.3) | 42.0 (38.6-45.3) | 45.0 (41.4-48.5) | 47.1 (43.3-50.9) | 18.3 (15.1-21.4) | 19.2 (16.7-21.7) | 26.9 (24-29.8) | 29.4 (26.3-32.4) | 31.5 (28.1-35) |
| 2016 | 22.2 (19-25.9) | 29.7 (27.1-32.5) | 40.8 (37.9-43.8) | 44.0 (40.3-47.1) | 51.1 (47.5-54.3) | 17.8 (14.9-20.7) | 21.8 (19.4-24.1) | 22.4 (20.1-24.7) | 27.0 (24.3-29.7) | 31.8 (28.7-34.9) |
| **Obese** |  |  |  |  |  |  |  |  |  |  |
| 2013 | 3.4 (1.2-5.7) | 8.0 (5.9-10.9) | 12.1 (9.5-14.6) | 21.3 (18.3-25.3) | 22.0 (18.9-25.6) | 5.5 (3.7-7.4) | 12.9 (9.9-15.9) | 15.6 (12.5-18.8) | 17.7 (14.3-21.1) | 20.7 (16.9-24.6) |
| 2014 | 5.7 (3.9-7.9) | 8.6 (7-10.7) | 12.8 (10.9-15.3) | 19.0 (16.3-21.9) | 23.7 (20.6-26.8) | 7.4 (5.4-9.4) | 12.8 (10.5-15.2) | 18.0 (15.6-20.5) | 21.3 (18.3-24.3) | 20.7 (17.4-24) |
| 2015 | 7.3 (5-9.5) | 9.7 (7.6-11.9) | 13.3 (11-15.7) | 19.1 (16.1-22.1) | 25.9 (22.4-29.5) | 9.6 (7.3-12) | 10.6 (8.4-12.8) | 13.9 (11.6-16.2) | 17.0 (14.5-19.5) | 16.9 (14.2-19.6) |
| 2016 | 6.7 (5.1-8.7) | 9.2 (7.1-11.2) | 14.1 (12-16.4) | 19.3 (16.8-22.6) | 23.0 (20.2-26.3) | 8.3 (6.4-10.1) | 12.2 (10.3-14) | 17.0 (14.7-19.3) | 18.4 (15.8-21.0) | 19.4 (16.7-22.1) |

**Supplementary Table 3a. Linear trends of abdominal obesity prevalence in women. Survey year 2013-2016 (N=32,799)**

|  | **Prevalence^a^ % (95 CI)** | | | |  |  |  |
| --- | --- | --- | --- | --- | --- | --- | --- |
|  | 2013  N=5,966 | 2014  N=8,223 | 2015  N=8,468 | 2016  N=10,142 | **Change 2013 to 2016^b^** | **P-value^c^** | **P-value of linear trend test^d^** |
| 18-29 (N=4,690) | 25.4 (20.9-29.8) | 28.1 (24.7-31.6) | 24.7 (21.4-28.0) | 25.9 (22.7-29.0) | 0.2 (-5.2; 5.6) | 0.93 | 0.75 |
| 30-39 (N=7,235) | 35.8 (31.8-39.8) | 34.6 (31.4-37.7) | 35.1 (32.0-38.3) | 36.4 (33.8-39.0) | 0.6 (-4.1; 5.3) | 0.79 | 0.73 |
| 40-49 (N=7,815) | 44.3 (40.5-48.0) | 48.3 (45.3-51.3) | 45.3 (42.2-48.4) | 44.2 (41.5-47.0) | -0.08 (-4.7; 4.5) | 0.97 | 0.66 |
| 50-59 (N=7,067) | 55.7 (51.8- 59.6) | 54.3 (51.1-57.5) | 57.2 (54.0-60.4) | 54.4 (51.4-57.5) | -0.9 (-5.9; 3.9) | 0.69 | 0.99 |
| 60-69 (N=5,992) | 63.3 (59.4-67.1) | 62.8 (59.3-66.2) | 63.3 (59.9-66.8) | 64.6 (61.5-67.7) | 1.4 (-3.4; 6.4) | 0.55 | 0.53 |

^a^ Waist circumference ≥80 cm for women

^b^Percentage point

^c^From the t-test

**Supplementary Table 3b. Linear trends of abdominal obesity prevalence in men. Survey year 2013-2016 (N=30,783)**

|  | **Prevalence^a^ % (95 CI)** | | | |  |  |  |
| --- | --- | --- | --- | --- | --- | --- | --- |
|  | 2013  N=5,796 | 2014  N=7,512 | 2015  N=8,078 | 2016  N=9,397 | **Change 2013 to 2016^b^** | **P-value^c^** | **P-value of linear trend test^d^** |
| 18-29 (N=3,775) | 10.0 (6.4-13.6) | 11.9 (9.2-14.5) | 13.6 (10.7-16.5) | 12.1 (9.5-14.6) | 2.3 (-2.0; 6.7) | 0.28 | 0.21 |
| 30-39 (N=6,504) | 18.1 (14.6-21.6) | 20.6 (18.0-23.2) | 21.5 (18.7-24.3) | 21.1 (18.6-23.6) | 3.0 (-1.2; 7.2) | 0.16 | 0.14 |
| 40-49 (N=7,360) | 32.8 (29.2-36.5) | 32.1 (29.1-35.1) | 35.8 (32.6-39.1) | 35.6 (32.7-38.4) | 2.2 (-2.3; 6.8) | 0.33 | 0.17 |
| 50-59 (N=6,608) | 51.8 (47.8- 55.7) | 49.8 (46.5-53.2) | 52.2 (48.6-55.8) | 50.4 (47.0-53.8) | -1.8 (-6.9; 3.3) | 0.49 | 0.69 |
| 60-69 (N=6,536) | 60.4 (56.7-64.1) | 61.1 (57.7-64.6) | 65.7 (62.1-69.2) | 65.3 (62.2-68.5) | 5.3 (0.4; 10.13) | 0.03 | 0.009 |

^a^ Waist circumference ≥94 cm for men

^b^Percentage point

^c^From the t-test
